# Outcome of Veno-Arterial Extracorporeal Membrane Oxygenation for Patients Undergoing Valvular Surgery

**DOI:** 10.1371/journal.pone.0063924

**Published:** 2013-05-23

**Authors:** Jian-Gang Wang, Jie Han, Yi-Xin Jia, Wen Zeng, Xiao-Tong Hou, Xu Meng

**Affiliations:** Department of Cardiac Surgery, Beijing Anzhen Hospital, Capital Medical University, Beijing, China; Università Vita-Salute San Raffaele, Italy

## Abstract

**Background:**

We evaluated retrospectively the early and midterm results of using veno-arterial extracorporeal membrane oxygenation (VA-ECMO) support in patients undergoing valvular surgery.

**Methods:**

A total of 87 patients undergoing valvular surgery received VA-ECMO due to refractory postcardiotomy cardiogenic shock (PCS), who were eligible for inclusion were enrolled in this study. Preoperative, perioperative, and postoperative variables were assessed and analyzed for possible associations with mortality in hospital and after discharge.

**Results:**

The mean age, additive EuroSCORE, and left ventricular ejection fraction (LVEF) for all patients was 65±7 years, 6.1±1.9 points, and 46% ±12%, respectively. The mean duration of VA-ECMO support was 61±37 hours. Intra-aortic balloon pumps (IABP) were implanted in 47.1% of patients. Weaning from VA-ECMO was successful in 59% of patients, and 49% were discharged. Multivariate analysis revealed that being >65 years old (odds ratio [OR], 2.75), receiving postoperative renal replacement treatment (OR, 2.47), having a peak lactate level ≥12 mmol L^–1^ (OR, 2.18), and receiving VA-ECMO for >60 hours (OR, 3.2) were independent predictors of in-hospital mortality. IABP support (OR, 0.46) was protective. In addition, persistent heart failure with an LVEF <40% was an independent predictor of mortality after discharge.

**Conclusions:**

VA-ECMO is an acceptable technique for the treatment of PCS in patients undergoing valvular surgery, who would otherwise die. It is justified by the good long-term outcomes of hospital survivors, but the use of VA-ECMO must be decided on an individual risk profile basis because of high morbidity and mortality rates.

## Introduction

Veno-arterial extracorporeal membrane oxygenation (VA-ECMO) is an established treatment option for adult patients with refractory cardiogenic shock that provides prolonged but temporary cardiac and respiratory support.[1], [2] Approximately 1% of patients who undergo routine cardiac surgical procedures experience refractory postcardiotomy cardiogenic shock (PCS) requiring prolonged postoperative hemodynamic support to allow recovery from reversible myocardial injury.[3], [4].

Valvular surgery patients, especially those with rheumatic disease, often have a long history of valve disease, abnormal hemodynamics, and severe decompensation of cardiac function.[5] These conditions can lead to poor left ventricular function with PCS. VA-ECMO can provide hemodynamic support that enables affected patients to recover from reversible myocardial injury.[6] Beyond its ability to provide biventricular support, VA-ECMO is attractive owing to its relative simplicity and low cost. Institutions that use VA-ECMO as a rescue therapy to treat PCS in patients undergoing valvular surgery need clear treatment protocols with defined therapeutic targets. Here, with the aims of creating such a protocol and thereby improving clinical outcomes, we provide a review of our experience with using VA-ECMO for the treatment of PCS in patients undergoing valvular surgery over a 7-year period.

## Materials and Methods

Each patient gave their informed written consent; the study protocol was conducted in accordance with the recommendations of the Declaration of Helsinki and was approved by (Institutional Review Board or Ethics Committee of Beijing Anzhen Hospital, Capital Medical University).

### Data Collection

Between January 2004 to December 2011, a total of 4,871 adult patients underwent valvular surgery at the Beijing Anzhen Hospital. Of these patients, 92 required VA-ECMO due to their inability to be weaned from cardiopulmonary bypass (CPB) (n = 37) or refractory PCS (n = 50). Only patients who received VA-ECMO for cardiac support (n = 87) were included in this retrospective study. Patients who received venovenous ECMO to treat postoperative pulmonary dysfunction (n = 5) were excluded.

VA-ECMO was instituted intra-operatively during the primary cardiac procedure or secondarily within 30 minutes of determining that the patient was suffering from delayed PCS. Secondary indications included progressive univentricular or biventricular forward or backward pump failure, intractable ventricular arrhythmia or fibrillation, or sudden idiopathic heart failure.

### VA-ECMO Device and Management

Indications for VA-ECMO support included the clinical criteria of PCS, including systolic arterial hypotension (<80 mmHg) and signs of end-organ failure, anaerobic metabolism, and metabolic acidosis (pH <7.3, lactate level >3.0 mmol/L, urinary rate <0.5 mL/kg) despite optimized supportive measures, such as intra-aortic balloon pumps (IABP), inotropes, nitric oxide and delivery of phosphodiesterase inhibitors. Hemodynamic criteria included a cardiac index of <1.8 L/m^2^ body surface area and pulmonary capillary wedge pressure(PCWP) of ≥20 mmHg.

The ECLS technique we employed has been described in detail elsewhere.[7] The VA-ECMO system (catalog no. CB1Q91R6; Medtronic, Inc., Anaheim, CA) was comprised of a centrifugal pump and a hollow-fiber microporous membrane oxygenator with an integrated heat exchanger. The femoral route for VA-ECMO support was preferred over the open sternotomy route because the presence of an open sternotomy wound increases the risk of bleeding and infection and makes postoperative nursing care more difficult.

The blood flow for VA-ECMO was calculated to supply at least adequate total systemic circulatory support (2.2 L min^–1^) and to achieve a mixed venous oxygen saturation (SvO_2_) level of 70%. The primary therapeutic goal of VA-ECMO was to achieve adequate hemodynamic support to provide sufficient perfusion of the patient’s vital organs. The use of inotropic agents was minimized to allow for optimal myocardial recovery while maintaining left ventricular ejection. (Inotrope score quantifies the amount of inotropic agents infused when hemodynamic support was applied.[7], [8])After 24 h of VA-ECMO support, heparin infusion was initiated to maintain activated clotting time in the range 160–180 s, depending on the patient’s risk of bleeding. Hematocrit levels were maintained at 30–35%. The typical settings were: a tidal volume of 8 mL kg^–1^, 8 breaths per minute, a positive end expiratory pressure of 10 cm H_2_O, a maximum ventilation pressure of 25 cm H_2_O, and a fraction of inspired oxygen (FiO_2_) of 0.4. Echocardiography was performed daily to assess cardiac recovery.

The criteria for weaning from VA-ECMO included SvO2 ≥70%, stable hemodynamics, absence of tamponade (determined by echocardiography), absence of left heart distention, and a left ventricular ejection fraction (LVEF) ≥40%. During weaning, blood flow was slowed to 0.5 L min^–1^ and vital signs were observed. If the patient’s hemodynamics remained stable, the VA-ECMO system was removed using intravenous anesthesia at the patient’s bedside, and primary repair of the patient’s vessels was undertaken. All patients underwent an echocardiogram immediately before being discharged from the hospital. All patients underwent regular follow-up assessments of their cardiac function in the outpatient department after hospital discharge.

### Statistical Analysis

Continuous data were expressed as means ± standard deviations and compared using Student’s *t*-tests or one-way analyses of variance (ANOVAs). Categorical variables were expressed as percentages and were evaluated using the chi-square or Fisher’s exact test. Kaplan-Meier survival curves were plotted to show survival trends, and survival data were compared with log-rank tests. A logistic regression model was used to evaluate the effect of various clinical parameters on survival, and odds ratios (ORs) were determined. Statistical significance was established at *p*<0.05. Data were analyzed using SPSS 12.0 statistical software (SPSS Inc., Chicago, IL).

## Results

### Patient Characteristics

The demographic characteristics and pre-VA-ECMO risk profiles of the 87 patients in the study cohort are presented in Table 1. Of these 87 patients, 54 (62%) were diagnosed with rheumatic valve disease, the most common reason for valvular surgery in our study. 51/87 patients (59%) presented with atrial fibrillation (AF). The mean LVEF for our study cohort was 46% ±12% and their mean EuroSCORE was 6.1±1.9.

**Table 1:** Patient demographic data and preoperative clinical status with respect to hospital outcomes.

Parameter	Total(n = 87)	Hospital survivors(n = 43)	Non-survivors(n = 44)	*p*-value
Age, y	65±7	58±12	67±8	0.009
Female (%)	41.3 (36)	32.6 (14)	50.0 (22)	0.129
NYHA class III-IV (%)	80.4	72.1 (31)	88.6(39)	0.062
Etiology, % (n)				
Rheumatic disease	62.1 (54)	48.8 (21)	75.0 (33)	0.015
Degenerative disease	19.5 (17)	25.6 (11)	13.6 (6)	0.186
Congenital disease	10.3 (9)	9.3 (4)	11.4 (5)	NS
Ischemia	8.1 (7)	9.3 (4)	6.8 (3)	0.713
Comorbidities, % (n)				
AF	58.6 (51)	30.2 (13)	86.4 (38)	<0.001
Hypertension	21.8 (19)	25.6 (11)	18.2 (8)	0.446
Diabetes	12.6 (11)	9.3 (4)	16.3 (7)	0.521
Renal failure	4.6 (4)	7.0 (3)	2.3 (1)	0.360
Stroke	10.3 (9)	16.3 (7)	4.5 (2)	0.089
Prior cardiac surgery, % (n)	12.6 (11)	11.6 (5)	13.6 (6)	0.521
Pulmonary hypertension, % (n)	48. 3(42)	53.5 (23)	43.2 (19)	0.394
Creatine kinase-MB level, U/L	48±27	49±19	47±21	0.573
Cardiac troponin T level, ng⋅m L^–1^	0.02±0.03	0.02±0.02	0.03±0.01	0.935
LVEF, %	46±12	49±17	43±13	0.090
EuroSCORE	6.1±1.9	3.7±1.7	8.1±1.4	<0.001

AF, atrial fibrillation; LVEF, left ventricular ejection fraction; NYHA, New York Heart Association functional class.

### VA-ECMO Support

The cardiac procedures undertaken and peri-operative clinical characteristics are summarized in Table 2. VA-ECMO was used in the operating room in 37/87 patients (43%) due to detection of circulatory instability during or immediately after weaning from cardiopulmonary bypass. A majority of the patients, 50/87 (57%), received hemodynamic support after primary cardiac surgery as a consequence of delayed PCS or for postoperative resuscitation in the intensive care unit. Application of the support system was successful in all 87 cases. The mean interval from the primary cardiac procedure to the initiation of hemodynamic support in these patients was 23.6 h; the interval was greater in non-survivors (27.1 h) than in hospital survivors (17.8 h, *p* = 0.042).

**Table 2:** Cardiac procedures and peri-operative clinical characteristics.

Parameter	Total(N = 87)	Hospital survivors(N = 43)	Non-survivors(N = 44)	*p*-value
MV surgery, % (n)	49.4 (43)	55.8 (24)	43.2 (19)	0.286
AV surgery, % (n)	23.0 (20)	20.9 (9)	25.0 (11)	0.800
AV and MV surgery, % (n)	25.3 (22)	30.2 (13)	20.5 (9)	0.332
TV repair, % (n)	39.1 (34)	34.9 (15)	43.2 (19)	0.512
RF ablation, % (n)	41.4 (36)	34.9 (15)	47.7 (21)	0.278
Combined CABG, % (n)	19.5 (17)	18.6 (8)	20.5 (9)	NS
Inotrope score^a^	22±6	18±5	27±5	<0.001
CPB time, min	182±93	179±91	183±34	0.713
Cross-clamp time, min	94±42	93±40	97±43	0.638
Blood cardioplegia (4°C), % (n)	43.6(38)	39.5(17)	47.7(21)	0.519
HTK solution (4°C), % (n)	56.4(49)	60.5(26)	52.3(23)	0.519
Peak creatine kinase-MB level, U/L	512±101	498±96	539±115	0.582
Peak cardiac troponin T level, ng⋅m L^–1^	23.50±12.87	22.94±10.63	26.32±15.13	0.438
Peak lactate level, mmol L^–1^	11.9±4.2	10.7±3.7	16.8±8.9	<0.001
IABP support, % (n)	47.1 (41)	65.1 (28)	29.5 (13)	<0.001
Duration of VA-ECMO, h	61±37	53±44	67±42	0.021
Mechanical ventilation, h	71±46	69±56	87±61	0.189
ICU stay, d	6.5±3.9	5.1±2.7	7.2±5.3	0.071

a Inotrope score = [doses of dopamine+dobutamine (in μg·kg^–1^·min^–1^)]+[doses of epinephrine+norepinephrine+isoproterenol (in μg·kg^–1^·min^–1^)]×100+[dose of milrinone (in μg·kg^–1^·min^–1^)]×15. Inotrope score quantifies the amount of inotropic agents infused when hemodynamic support was applied^7,8^.

AV, aortic valve; CABG, coronary artery bypass grafting; CPB, cardiopulmonary bypass; ICU, intensive care unit; MV, mitral valve; RF, radiofrequency; TV, tricuspid valve.

**Figure 1:**
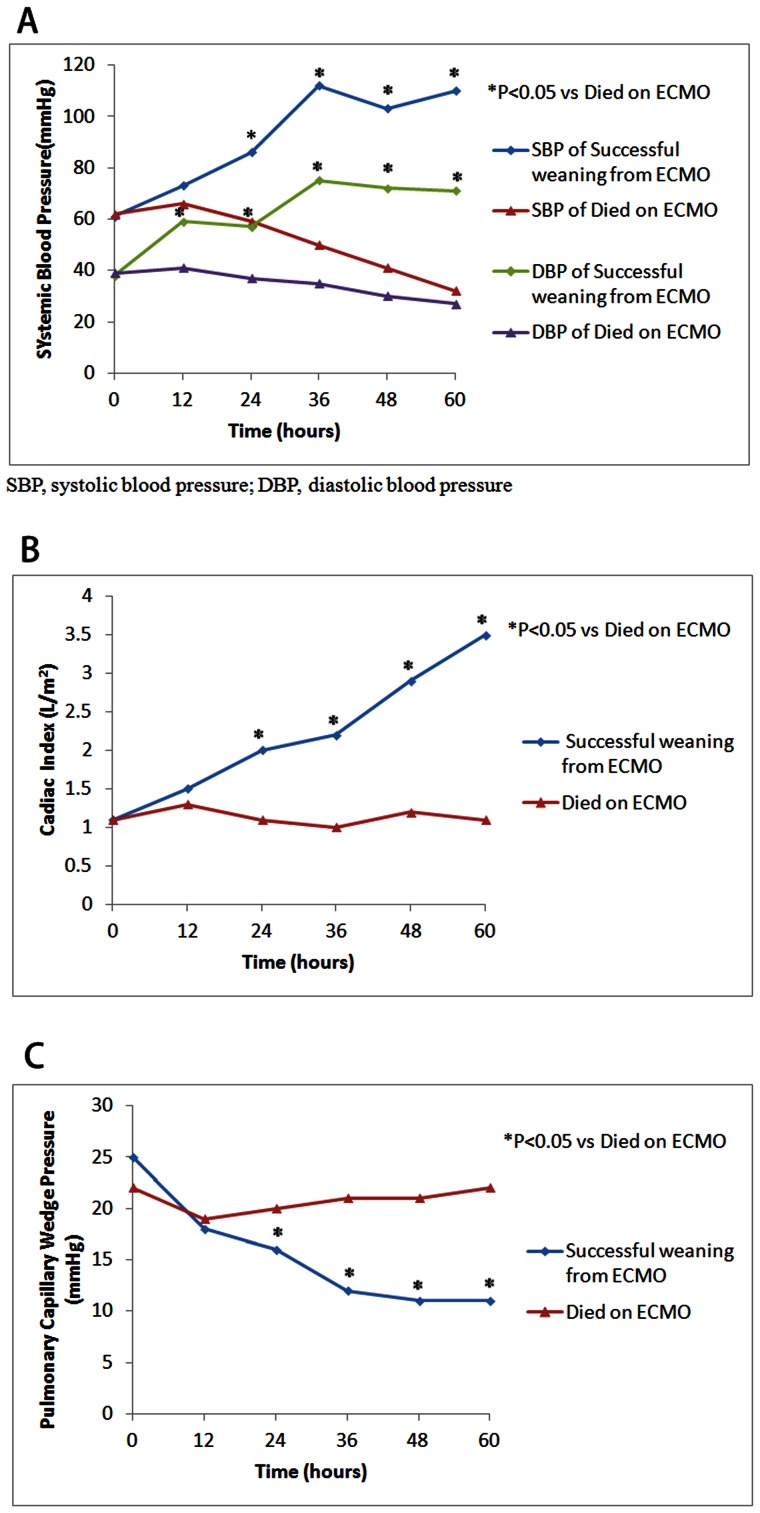
Blood pressure (A), cardiac index (B), and pulmonary capillary wedge pressure (C) changes curves in patients successful weaning from VA-ECMO and died on VA-ECMO.

Successful weaning from VA-ECMO was possible in 51/87 patients (59%). However in 36 patients (41%) whom total systemic circulatory supports and SvO_2_ could not achieved the targets, cardiac function did not improve and weaning was impossible, VA-ECMO support had to be withdrawn, and they subsequently died. Figure 1 shows the hemodynamic parameter, including systemic blood pressure, cardiac index and PCWP improved significantly after VA-ECMO implementation in patients successful weaning from the VA-ECMO (*p*<0.05).

Of the 51 patients who were weaned successfully from support, 43 (49%) were subsequently discharged from hospital after a mean period of 43.4±12.8 days. However, the remaining 8 weaned patients developed multi-organ failure and then died in the hospital. The primary cause of death in these 8 patients was sepsis with consecutive multi-organ failure. These complications occurred more often in patients aged over 75 years and in those with comorbidities, such as diabetes, chronic obstructive pulmonary disease and renal insufficiency. The overall in-hospital mortality rate was 51%.

The main cause of death in patients who could not be weaned from support was persistent heart failure without any improvement in cardiac function (22/36 patients; 61%). Other causes of death were sepsis with consecutive multi-organ failure (10/36; 28%), disseminated intravascular coagulation (3/36; 8%), and cerebral infarction and bleeding (1/36; 3%).

### Peri-operative Procedures and Complications

Overall, 60 (69.0%) of the 87 patients given PCS-related and VA-ECMO-related complications. The most common complication was PCS-related renal failure requiring continuous venovenous hemofiltration (e.g. renal replacement therapy), followed systemic infection defined by a positive blood culture (Table 3). Gram-positive *cocci infections were observed in 9 patients and* Gram-*negative bacilli infections were observed in 4 (other) patients.* Low frequency VA-ECMO-related complications included limb ischemia, neurological complications caused by cerebral stroke (N = 4) or hemorrhage (N = 2), and limb amputation due to ischemia (Table 3). When average blood pressure in the dorsal pedal artery fell below 40 mmHg, a distal leg perfusion cannula (8-F or 10-F) was introduced (N = 37). All patients who suffered limb ischemia had been given direct peripheral cannulation for VA-ECMO.

**Table 3:** Summary of postoperative clinical events and complications.

Postoperative clinical event/complication	Total(n = 87)	Hospital survivors(n = 43)	Non-survivors(n = 44)	*p*-value
Postoperative drainage loss (mL)	1317±873	1221±534	1386±825	0.934
Repeat thoracotomy, % (n)	16.1 (14)	18.6 (8)	13.6 (6)	0.771
RBC transfusion (units)	21.4±8.9	20.0±6.3	21.9±8.6	0.386
Renal replacement therapy, % (n)	25.3 (22)	14.0 (6)	36.4 (16)	0.025
Infection, % (n)	14.9 (13)	16.3 (7)	13.6 (6)	0.772
Lower limb ischemia, % (n)	5.7 (5)	7.0 (3)	4.5 (2)	0.676
Neurological complications, % (n)	6.9 (6)	9.3 (4)	4.5 (2)	0.434
Leg amputation, % (n)	1.1 (1)	0	2.3 (1)	NS

RBC, red blood cell.

### Univariate Analysis of the Risk Factors for In-hospital Mortality

The results of univariate comparisons of demographic data, operative and VA-ECMO-related data, and complication data between survivors (weaned and discharged) and non-survivors (dead before discharge) are reported in Tables 1, 2, and 3, respectively. As reported in Table 1, compared to the non-survivors, the survivors were significantly younger (*p* = 0.009), had a lower incidence of rheumatic valve disease (*p* = 0.015) and AF disease (*p*<0.001), had a lower EuroSCORE value (*p*<0.001), a lower inotrope score (*p*<0.001), a lower level of peak lactate level (*p*<0.001), a shorter duration of VA-ECMO (*p* = 0.021), a lower incidence of renal replacement therapy after surgery (*p* = 0.025), and a higher incidence of IABP support (*p*<0.001).

### Multivariate Analysis of the Independent Risk Factors of In-hospital Mortality

Four independent risk factors remained statistically significant by multivariate logistic regression analysis adjusted for imbalances at baseline: age >65 years (adjusted OR = 2.75, *p* = 0.02, 95% confidence interval [CI]: 1.12–1.46), requirement for renal replacement treatment (adjusted OR = 2.47, *p* = 0.006, 95% CI: 1.48–4.13), peak lactate level ≥12 mmol L^–1^ (adjusted OR = 2.18, *p*<0.001, 95% CI: 1.95–3.49), and failure to be weaned from VA-ECMO after 60 hours (adjusted OR = 3.2, *p*<0.001, 95% CI: 1.2–10.8). IABP support (adjusted OR = 0.46, *p*<0.001, 95% CI: 0.29–0.68) was found to be a protective factor. (Table 4).

**Table 4:** Logistic regression model to identify parameters associated with in-hospital mortality: odds ratio (OR), 95% confidence intervals (CI) and p-values.

	Survival/In-hospital death (Crude)			Survival/In-hospital death (Adjusted)		
	OR	95.0% CI	*p*-value	OR	95.0% CI	*p*-value
Age>65 years old	2.98	(1.28, 3.08)	0.007	2.75	(1.12, 1.46)	0.020
Peak lactate level ≥12 mmol L^–1^	2.82	(2.13, 4.16)	<0.001	2.18	(1.95, 3.49)	<0.001
Receiving postoperative renal replacement treatment	2.93	(1.89, 4.83)	<0.001	2.47	(1.48, 4.13)	0.006
Receiving VA-ECMO for >60 hours	4.1	(2.0, 12.4)	<0.001	3.2	(1.2, 10.8)	<0.001
IABP support	0.42	(0.67, 0.93)	<0.001	0.46	(0.29, 0.68)	<0.001

### Follow-up

**Figure 2:**
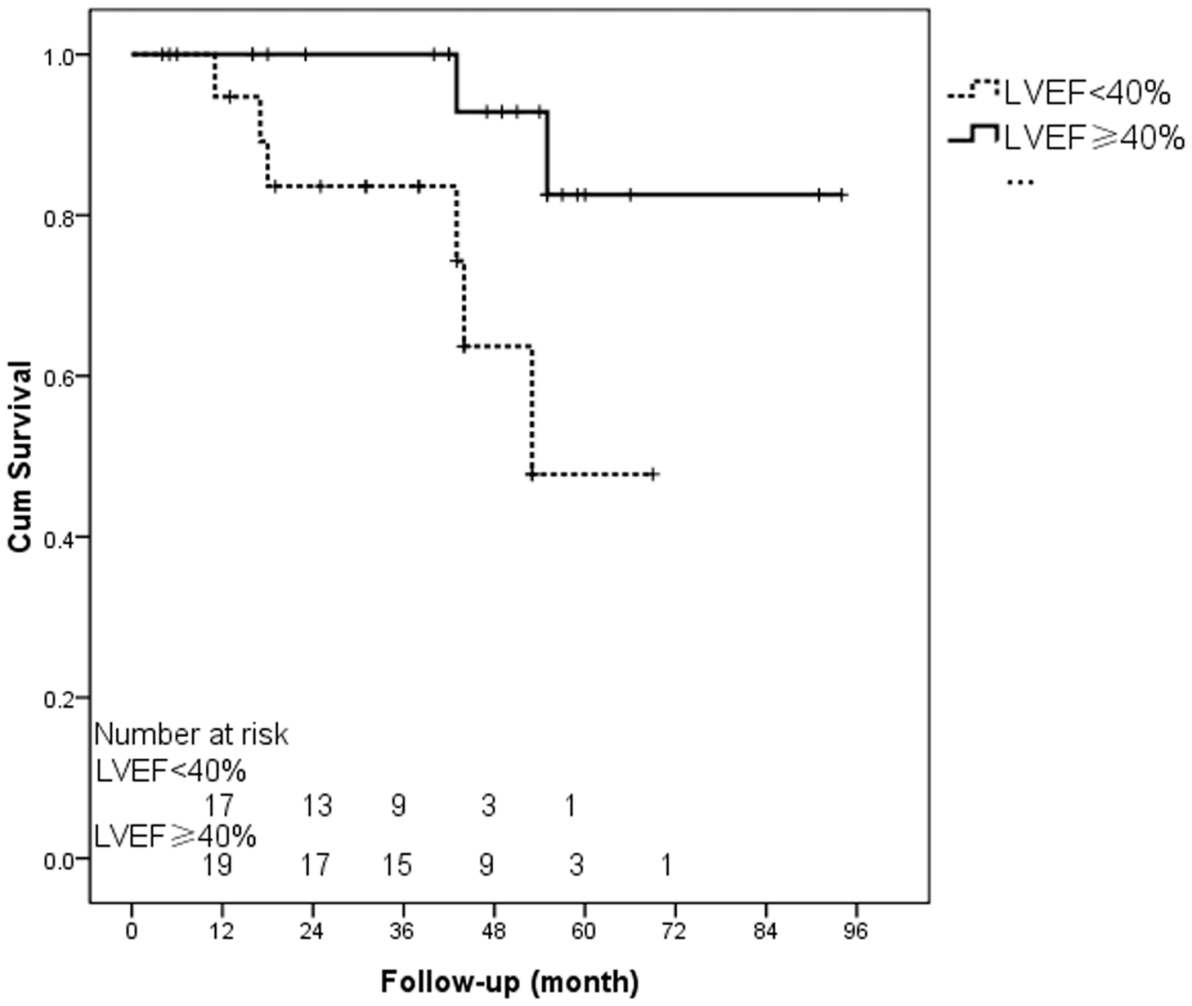
Kaplan-Meier plot of survival curves for patients with a postoperative LVEF of ≥40% or <40% (log-rank test, *p*<0.019).

The mean, median, and range of the follow-up time were 24.6±22.7, 16.8 and 1–94 months, respectively. Survival analysis by the Kaplan-Meier method showed that the overall cumulative survival rate among patients who were withdrawn successfully from VA-ECMO and discharged from the hospital was 92% ±4% after 1 year and 66% ±11% after 5 years. Of the eight late deaths (after hospital discharge), six were considered to be cardiac-related, one was due to encephalorrhagia, and one was recorded as an unknown etiology. A postoperative LVEF of <40% before hospital discharge was the only independent risk factor for late death (hazard ratio [HR]: 8.7; 95% CI: 3.7–16.4; *p*<0.001). Figure 2 shows the Kaplan-Meier curve for survival in patients with a postoperative LVEF of <40% or ≥40% (log-rank test, *p* = 0.019). The estimated survival time of patients with a postoperative LVEF of <40% was 52.6±5.2 months.

## Discussion

Our retrospective study examining PCS in valvular surgery patients at a single institution showed that a survival benefit was conferred by hemodynamic support with VA-ECMO. A logistic regression model identified age of at least 65 years, the need for postoperative renal replacement treatment, a peak lactate level ≥12 mmol L^–1^ and the need for more than 60 hours of VA-ECMO were independent predictors of in-hospital mortality. Conversely, IABP support emerged as a protective factor. Finally, a postoperative LVEF <40% before hospital discharge was an independent predictor of mortality after hospital discharge.

Valvular surgery patients, especially those with rheumatic disease, often have a long history of valve disease, abnormal hemodynamics, and severe decompensation of heart function.[5] These conditions can lead to poor left ventricular function with PCS. Given this dismal prognosis, all treatment options must be considered carefully. VA-ECMO is a promising technique because it provides active circulatory support to patients with severe cardiac failure following cardiopulmonary bypass who are unresponsive to inotropes and/or an IABP alone.[2], [3], [6], [9], [10] Use of VA-ECMO has continued to increase, particularly over the last 5 years with the expansion of valvular surgery. In our institution, VA-ECMO is preferred over other assist devices because of its versatility. The better in-hospital survival rate observed in patients treated with VA-ECMO in the present study provides strong evidence that VA-ECMO can facilitate recovery and improve outcome among patients with PCS and valvular disease.

Importantly, early provision of hemodynamic support may prevent the myocardial damage that can be caused by inotropic agents or hypoxia. Moreover, hemodynamic support allows patients to recover from myocardial injury over an extended period of time. There is no established consensus for the optimal timing of VA-ECMO. However, it is noteworthy that, in this study, the time elapsed before VA-ECMO commencement was significantly shorter in survivors than in non-survivors (*p* = 0.042). This finding is consistent with the notion that earlier VA-ECMO may reduce mortality risk. Moreover, based on our experience, we believe that valvular surgery patients with severe left ventricular dysfunction may benefit from receiving VA-ECMO intra-operatively or very early in the postoperative period.

Based on the experience of physicians at our institution, we now consider VA-ECMO therapy to be a valuable option for the treatment of myocardial infarction and low output syndrome in cardiac procedures perioperatively. The present finding that VA-ECMO was associated with improved in-hospital survival is consistent with the results of prior studies reporting survival rates in the range of 45–67% in valvular surgery patients with PCS who underwent VA-ECMO.[6], [7], [11], [12] It is our view that VA-ECMO may play a crucial role in the successful treatment of PCS in patients with valve disease; these patients might benefit more if VA-ECMO is started intra-operatively, or very early in the postoperative course, to prevent clinical deterioration.

Increased peak lactate level, which reflects persistent anerobic metabolism and severe tissue acidosis, was the strongest predictor of mortality. Logistic regression analysis of the present data revealed that a peak lactate level of ≥12 mmol L^–1^ before provision of hemodynamic support predicted increased mortality, consistent with our previous findings.[7] These findings also suggest that early support, before lactate levels become precarious, could improve outcomes in patients presenting with PCS.

The major disadvantage of VA-ECMO is the need for anticoagulation and large amounts of transfused blood products, which may intensify systemic inflammatory responses induced by the initial surgery, support components, and PCS itself.[12]–[15] Systemic heparinization is still advisable in patients with no elevated risk factors for bleeding because of the risk of end organ damage from microthrombus and fibrin deposition, though the level required is still being debated.[13].

The use of an IABP was a predictor of survival. Patients with IABPs were more likely to be weaned than those without IABPs, perhaps owing to the beneficial effects of afterload reduction on myocardial recovery, better coronary flow, or improved organ function with pulsatile flow.[16] Our findings support the recommendation that all patients that require VA-ECMO support for cardiac failure have concomitant IABP support.

Renal failure and the need for hemofiltration was the most common complication observed in our study population. Decreased renal reserve capacity and intolerance to hypoxia and hypoperfusion may be the main reasons for the high incidence of renal failure in rheumatic valve disease patients.[7] The predisposing factors for renal failure could also include blood loss, sepsis and drug toxicity. Patients in this study who survived recovered renal function as their cardiac function improved, and no survivors examined during the follow-up period needed long term dialysis.

Infection was another common complication which produced mediators of inflammation that led to multiorgan failure and ultimately death. This is likely due to several factors related to both the patient and the medical therapy. VA-ECMO patients require invasive procedures, are frequently exposed to broad-spectrum antibiotics, and require the prolonged use of invasive support devices, such as central lines, urinary catheters, and endotracheal tubes. Certain factors inherent to VA-ECMO support may also contribute to the high rate of acquired infections in this population.[17].

Despite high in-hospital mortality, the long-term survival of patients discharged from hospital after VA-ECMO for PCS was good: 92% ±4% at 1 year and 66% ±11% at 5 years. These data are comparable with the experiences reported for VA-ECMO populations at the Cleveland Clinic and the Heart Center of Leipzig University, where patients surviving 30 days had a 63–74% chance of survival after 5 years.[1], [11].

The postoperative LVEF value was the only independent risk factor for death after hospital discharge. Cardiac surgery may improve the LVEF by recruiting the hibernating myocardium, or may worsen LVEF due to inadequate intra-operative myocardial protection.[4] Regardless, impaired LVEF postoperatively is an ominous sign. Close follow-up and early intervention, using either a ventricular-assist device or an urgent listing for cardiac transplantation, appears to be critical for the future survival of these patients.

The limitations of this study were the moderate number of patients included and the retrospective design. Potential confounding factors, which are difficult to control for, may have been present. Outcomes may be improved by further refinement of surgical techniques and increasing clinicians’ experience with VA-ECMO. Further laboratory investigations and prospective clinical studies will lead to improvements in therapeutic protocols for valvular surgery patients with PCS.

In summary, the present findings indicate that the VA-ECMO is a suitable alternative for patients with PCS complicated with valvular disease. Because the prognosis of patients receiving hemodynamic support is affected by the severity of PCS at the time of implantation, it would be prudent to apply hemodynamic support without delay when it is a reasonable option. Of course, good general postoperative care, proper organization and implementation should be emphasized to prevent the complications of VA-ECMO and to improve the outcomes.
